# Protein Folding and Aggregation into Amyloid: The Interference by Natural Phenolic Compounds

**DOI:** 10.3390/ijms140612411

**Published:** 2013-06-13

**Authors:** Massimo Stefani, Stefania Rigacci

**Affiliations:** 1 Department of Experimental and Clinical Biomedical Sciences, University of Florence, Viale Morgagni 50, Florence 50134, Italy; E-Mail: stefania.rigacci@unifi.it; 2 Research Centre on the Molecular Basis of Neurodegeneration, Viale Morgagni 50, Florence 50134, Italy

**Keywords:** amyloid, amyloid aggregation, polyphenols, natural phenols

## Abstract

Amyloid aggregation is a hallmark of several degenerative diseases affecting the brain or peripheral tissues, whose intermediates (oligomers, protofibrils) and final mature fibrils display different toxicity. Consequently, compounds counteracting amyloid aggregation have been investigated for their ability (i) to stabilize toxic amyloid precursors; (ii) to prevent the growth of toxic oligomers or speed that of fibrils; (iii) to inhibit fibril growth and deposition; (iv) to disassemble preformed fibrils; and (v) to favor amyloid clearance. Natural phenols, a wide panel of plant molecules, are one of the most actively investigated categories of potential amyloid inhibitors. They are considered responsible for the beneficial effects of several traditional diets being present in green tea, extra virgin olive oil, red wine, spices, berries and aromatic herbs. Accordingly, it has been proposed that some natural phenols could be exploited to prevent and to treat amyloid diseases, and recent studies have provided significant information on their ability to inhibit peptide/protein aggregation in various ways and to stimulate cell defenses, leading to identify shared or specific mechanisms. In the first part of this review, we will overview the significance and mechanisms of amyloid aggregation and aggregate toxicity; then, we will summarize the recent achievements on protection against amyloid diseases by many natural phenols.

## 1. Introduction

The cellular genome encodes not only the amino acid sequences and the specific three-dimensional structures of proteins associated with their biological functions, but also the way these structures are attained through protein folding, a self-organization process, whose information is completely contained within the primary structure of the polypeptide chain. In the last 10–15 years, a combination of theoretical and experimental studies has unraveled many key features of protein folding, the mechanisms responsible for its control in living systems and the key role of these mechanisms in maintaining cell wellness and tissue functionality [1]. Such information has raised a number of important new issues. For example, it has become clear that protein folding and unfolding are intrinsically associated with several cellular processes, including molecular trafficking within or between cells and tissues, crossing of cell membranes, cell cycle regulation and immune responses. Such observations have led to conclude that any failure of a specific protein/peptide to fold/unfold correctly or to remain properly folded (misfolding) is at the basis of different types of biological malfunctions, resulting in various types of diseases most often characterized by the presence of deposits of ordered protein aggregates in the affected tissues [2].

It is well established that “misfolded” proteins, *i.e.*, proteins partially or completely devoid of the conformation associated with optimal stability and biological function, are more or less inactive, thus explaining the “loss-of-function” features of the associated pathologies; however, this process can also lead misfolded polypeptide chains to gain a “toxic” conformation, which enables them to aggregate into ordered polymeric assemblies and/or to interact inappropriately with other cellular components, impairing cell viability and function and, eventually, resulting in cell death [3,4]. Therefore, the molecular basis of these “protein misfolding diseases”, or “conformational diseases”, may ultimately be traced back to the presence, in a living system, of protein or peptide molecules with incorrect structures, different from those found in normally functioning organisms [5–7]. The most important subset of protein misfolding diseases includes conditions whose molecular basis can be traced back to a toxic “gain-of-function”, whereby specific peptides/proteins in their unstable, misfolded state self-organize into insoluble, cytotoxic, ordered and stable fibrillar polymeric assemblies with shared conformational features (amyloid aggregates) [8].

Indeed, protein misfolding and its frequent outcome, aggregation, is one of the most exciting new frontiers in protein science, cell biology and molecular medicine. Presently, it is thought that protein folding and aggregation are determined by similar physicochemical properties and, hence, are competing processes [9–11]; accordingly, a better knowledge of the molecular basis of protein misfolding and aggregation may help to elucidate the structural determinants and the physicochemical features of protein folding and *vice versa*. However, it is also expected to shed light on the molecular and biochemical basis of a number of pathological conditions, some of which with severe medical and social impact, such as Alzheimer’s disease (AD) and Parkinson’s disease (PD), type 2 diabetes, cystic fibrosis, some forms of emphysema, familial hypercholesterolemia and others, for which effective therapy is presently lacking [3,4]. Such information will provide clues to develop possible therapeutic strategies suitable to hinder growth and deposition or to favor removal of amyloid aggregates or their precursors, as well as to foster the cell defenses against amyloid toxicity.

In amyloid diseases, the failure of specific peptides or proteins to fold or to remain correctly folded triggers aggregate nucleation, the first step of a process eventually giving rise to fibrillar amyloid deposits in tissue. The involved polypeptides may include full length proteins, biological peptides or peptide fragments arising from proteolytic processing of larger precursors [3,4]. Usually, the aggregated peptides/proteins display wild-type (wt) sequences, as it happens in the sporadic forms of the diseases; however, in several cases, these can also be variants resulting from genetic mutations associated with early-onset familial forms of the diseases. Even though these familial forms are much rarer than the sporadic counterparts and may possibly represent distinct pathologies, their study, both in human and in transgenic animal models, has provided a valuable knowledge on the molecular basis of the much more widespread sporadic forms (reviewed in [12]). For example, a strong association has been shown between the age of the onset of the familial forms and the increase of the aggregation propensity of the mutated proteins. Such findings also support the widely accepted link between protein aggregation and clinical manifestations of the disease (reviewed in [13,14]). However, the structure of the pathogenic species, the molecular basis of their toxicity to cells and the contribution of the latter to the overall derangement of tissue function are still the subject of intense debate [15–21].

In many systemic and CNS amyloidoses, such as AD and Creutzfeldt-Jakob diseases, light chain, serum amyloid-A (SAA), dialysis-related amyloidoses and type-II diabetes, the amyloid deposits are mainly extracellular and appear as thread-like fibrillar structures, sometimes assembled further into larger aggregates or plaques. Yet, in systemic amyloidoses, other components, such as collagen, glycosaminoglycans and proteins (e.g., serum amyloid-P (SAP)), are often present in the deposits protecting them against degradation [22–24]. However, other conditions (AD, PD and poly(Q) diseases, amyotrophic lateral sclerosis (ALS)) are characterized by intracellular protein deposits with basic structural features similar to those shown by the extracellular deposits. Intracellular deposits are found either in the cytoplasm, in the form of specialized structures, known as aggresomes, in inclusion bodies, such as Lewy, Bunina or Russell bodies, or in the nucleus and often arise from deficits in the cell’s protective machineries.

Until the end of 1990s, the idea that mature amyloid fibrils are the key responsible for cell impairment and tissue damage provided a theoretical frame to rationalize the molecular basis of the known amyloid diseases, as well as to explore therapeutic approaches to amyloidoses mainly aimed at hindering growth and deposition of fibrillar aggregates. This view was first questioned in 1998 [15], and subsequently, an impressive body of experimental data showed that the aggregation nuclei and pre-fibrillar assemblies transiently arising in the path of fibrillization of several peptides and proteins and preceding the appearance of mature fibrils are the main toxic entities [16–20,25–29]. The increasing importance of these pre-fibrillar aggregates stems from the fact that soluble oligomers comparable to those appearing at the onset of fibrillization of several peptides and proteins have been repeatedly detected in and purified from, cultured cells and animal tissues, where the monomeric precursors are expressed [27–30]. These data have led to establish that amyloid oligomers are really formed *in vivo*, not only in cultured cells, but also in diseased tissue, both in animal models and in humans, providing convincing proof that they are really associated with cell/tissue impairment, directly contributing to it.

The awareness that mature amyloid fibrils are substantially devoid of cytotoxicity does not imply that they are substantially innocuous. Actually, the huge burden of deposited material in tissue in several systemic amyloidoses may by itself damage organs simply by hindering a proper flow of nutrients to the cells [23]. However, such a notion has led to re-consider, at least in part, the targets of the pharmacological strategies aimed at treating the various amyloidoses; in fact, the latter have been modified in order to search for molecules able to reduce the accumulation of misfolded proteins, as well as their assembly into toxic pre-fibrillar aggregates, rather than at simply hindering their eventual growth into mature fibrils. Nevertheless, in some cases, mature amyloid fibrils can affect directly cell viability [31–33], whereas in other cases, they can be a source of toxic pre fibrillar species [34,35].

The different assemblies, from small oligomers to the highly polymeric mature fibrils, appearing in the fibrillization process, as in path intermediates or off-path final products, differ remarkably not only in their cytotoxic potential, but also in the cellular mechanisms and functions they interfere with. For instance, Aβ42 oligomers found in the brains of AD people impair long-term potentiation [17,36] and raise endoplasmic reticulum stress [37] and mitochondria derangement [38], whereas the fibrillar Aβ deposits appear to be mainly responsible for the tissue neuroinflammatory response [39,40]. Moreover, in the case of the Aβ peptides, aggregate cytotoxicity is likely to depend on the aggregation state-specific uptake, oligomer toxicity appearing to be associated with oligomer internalization by endocytosis [41].

The idea that mature fibrils are substantially inert, harmless deposits of the toxic precursors implies that their growth has the significance of a cell defense mechanism and contributes toward explaining the lack of direct correlation between the load of amyloid plaques in human AD brain and the severity of the clinical symptoms [42]. However, up until now, the growing information on the effects of amyloids on cell biology has not resulted in any unifying model describing, for all forms of amyloidoses, the molecular basis of protein aggregation at physiological conditions, the identity of the aggregated species responsible for cell/tissue functional and viability impairment *in vivo* and the molecular mechanism(s) of the latter. In addition, in spite of their recognized importance as main cytotoxic entities, a severe lack of information still remains on the structural features of the oligomeric assemblies arising transiently in tissue. Actually, at present, it is only recognized that amyloid oligomers arising as in-path or off-path intermediates of amyloid fibril growth display increased flexibility and an exposed hydrophobic surface with respect to the parent natively folded monomers and their final product, mature fibrils. However, in general, these species, when grown at differing environmental conditions from the same peptide/protein, can exhibit remarkably variable conformational and biophysical features and cytotoxicity, eventually generating fibrils with different properties. For this reason, oligomer/fibril polymorphism and its effect on biological systems is a theme of increasing interest in amyloid science.

The search for any therapeutic strategy against amyloid diseases requires a deep knowledge of the biophysical and structural determinants of peptide/protein aggregation, of the pattern of aggregate growth, of the identification of the species that are responsible for cell/tissue derangement and of the mechanisms and molecular basis of the latter. This paper reports some of these issues, providing a review of the main data supporting experimentally the anti-aggregation and protective effects of many investigated organic and peptidic compounds, with particular emphasis to natural phenolic substances, whose anti-aggregation and beneficial properties will be covered in the second part of the review.

## 2. Amyloid Aggregation: Overview and Mechanisms

In general, protein aggregation into pre-fibrillar and fibrillar assemblies results from increased synthesis, reduced clearance, specific mutations, misprocessing, proteolysis or deficits in the cellular mechanisms aimed at managing misfolded species and causes derangement of the cellular proteostasis [43]. Presently, it is believed that, at least in most cases, both the functional conformation of a protein and that leading to aggregation into amyloid fibrils arise from shared precursor(s) generated in the folding process and/or arising from some of the states in dynamic equilibrium with the folded one. Partially folded states may also appear as a consequence of partial denaturation of the native state, resulting from misprocessing, specific mutations, any perturbation of the microenvironment (temperature, pH, ionic strength, pressure, shear forces, metal ion or macromolecular concentration) or any increase of the concentration of the nucleation precursors above a critical threshold (reviewed in [2]). Under these conditions, the partially unfolded molecules display altered tertiary structures, while retaining or even increasing the secondary one, as it is the case of some natively unfolded proteins that temporarily acquire partially folded conformations [44,45], thus undergoing more or less heavy structural reorganization and self-assembly into fibrils [3,4]. In such an ensemble of unfolded or partially folded states, hydrophobic groups and main chain amides, normally shielded into the compactly folded native state, become exposed to the solvent and can establish new intermolecular interactions that foster subsequent growth of large protein aggregates [4]. A balance must therefore exist between protein folding and misfolding that normally favors the former and whose alteration can contribute to aggregate appearance.

A protein can be shifted from the folding to the aggregation side of its energy landscape by enhancing the factors that more consistently promote aggregation over-folding. In most cases, protein aggregation may be triggered by mild destabilization of the medium conditions (a moderate shift of the temperature/pH or the presence of low amounts of denaturing agents or of co-solvents) that may increase the stability of the secondary contacts, while reducing that of the tertiary interactions in the folded protein [46–48].

The generation of aggregation nuclei at the onset of protein aggregation is considered the rate-limiting step of the latter, accounting for the delay times of polymer appearance both *in vitro* and, possibly, *in vivo*. However, at variance with protein folding, presently, much less is known on the conformational states available to an aggregating polypeptide chain and on the structural features at the atomic level of the early oligomeric assemblies; several reasons account for this, including oligomer instability and broad heterogeneity. In some cases, instead of aggregation nuclei, spherical oligomers and other pre-fibrillar forms, including curvy protofibrils, apparently resulting from a nucleation-independent path, can be formed in the absence of any lag phase [49–52]. Often, it is not clear the difference between oligomers and aggregation nuclei nor whether oligomers are on-pathway products, growing by direct recruitment of monomers or off-pathway dead-end reversible intermediates [51,53–55]. Finally, a protein can also aggregate by initially populating monomeric or oligomeric states, where it substantially maintains its natively folded structure before undergoing structural rearrangements into amyloids [56–61].

As stated in the Introduction, present knowledge considers the early pre-fibrillar species arising in the path of amyloid aggregation those endowed with the highest toxicity to cells. A step forward in understanding the molecular basis of amyloid toxicity was made in 2002, when it was first shown that, similarly to the tendency to aggregate, also amyloid cytotoxicity could not be considered a specific property of the few peptides and proteins found aggregated in amyloid diseases, possibly arising from some peculiarity of their specific amino acid sequences; rather, aggregate cytotoxicity appeared as a generic feature of the common basic amyloid fold shared by aggregates grown from any peptide/protein [20]. Such a knowledge provided further significance to the concept of life on the edge between the competing folding and aggregation processes and, hence, between function and dysfunction; it also highlighted the importance of the structural and functional adaptations set up by protein evolution to favor the functional side of such a border [62]. Actually, the view of protein aggregation as a property inherent to the backbone of any polypeptide chain suggests the existence of a previously unconsidered constraint in protein evolution; indeed, the latter must have discarded every functional sequence with any significant tendency to aggregate under the conditions where its biological function is carried out. The generic tendency of polypeptide chains to aggregate also underscores the biochemical importance and the biological significance of the complex molecular machineries responsible for the quality control of protein folding and for proteostasis. The latter include the molecular chaperones, the ubiquitin-proteasome pathway of protein degradation, the heat shock response in the cytosol and the unfolded protein response in the ER (reviewed in [63,64]). The evolution of such a complex biochemical machinery, as well as of other adaptations aimed at reducing the tendency of natural proteins to aggregate, testifies to the key importance, for cells, to avoid the appearance and build-up of any potentially aggregating protein or peptide.

The studies performed in the last few years have established new milestones in the theme of protein misfolding and aggregation, providing new clues to the development of effective anti-aggregation drugs. Presently, there is increasing awareness that protein aggregation is much more widespread than previously believed either in biology or in medicine. Actually, it can expected that the number of recognized pathological conditions with amyloid deposits will increase in the future, as has happened in recent years [12]. In some cases, amyloids with physiological significance have also been described (reviewed in [65]).

## 3. Amyloid Aggregates: Mechanisms of Cytotoxicity

The possibility to develop effective therapeutic or preservation tools to combat amyloid toxicity requires a better understanding of the key features of cell/tissue derangement upon exposure to toxic amyloids. Deciphering the latter in its complexity and self-reinforcing effects can be quite demanding; nevertheless, some basic shared perturbations in the exposed cells have been clearly described. The presence of intra- or extra-cellular toxic aggregates can impair a number of cell functions, from synaptic transmission and plasticity to cell membrane permeability, mitochondria functioning, ER homeostasis, nuclear transcription and cell signaling, eventually leading to cell death by apoptosis or, less frequently, by necrosis [66–71]. However, in most cases, initial perturbations of fundamental cellular processes appear to underlie the impairment of cell function induced by aggregates of disease-associated polypeptides. Increasing information points to a central role performed by alterations of the intracellular redox status, synaptic transmission, mitochondria efficiency and free Ca^2+^ levels in cells exposed to toxic aggregates [72–79]; other causes, such as transcriptional derangement, have also been proposed [68,80].

That oxidative stress plays a key role in cell sufferance following exposure to the early aggregates is stressed by many experimental data [80–82]. Cell treatment with antioxidants, such as tocopherol, lipoic acid, reduced glutathione or phenolic substances, is protective against aggregate toxicity [83–86]. Data also point to direct effects of ageing and oxidative stress on cell viability in nerve tissue through a reduction of the activity and expression levels of the proteasome [87,88], whose engulfment or inhibition are likely to result in the accumulation of oxidized or otherwise damaged or misfolded proteins, thus increasing the detrimental effects of reactive oxygen species (ROS) [89]. In this regard, a possible role of Hsp27 in preventing polyglutamine cytotoxicity by suppressing ROS production has been proposed in cells transiently transfected with vectors expressing exon 1 to produce varying lengths of glutamine repeats in huntingtin (htt) [90,91].

It is not yet clear why protein aggregation is followed by increased ROS production either in the cell or *in vitro*. Specific mechanisms may be involved, as in the case of Aβ42 [92] and α-synuclein (α-syn) in dopaminergic neurons in the substantia nigra [93]. The generation of hydroxide radicals from hydrogen peroxide by metal ions, such as Fe, Cu and Zn, has also been proposed as a cause of oxidative stress [94,95], together with an upregulation of membrane enzymes producing hydrogen peroxide (NADPH oxidase, cytochrome P450 reductase [96,97]). More generally, intracellular oxidative stress can be related to some form of destabilization of cell membranes by toxic species with loss of regulation of plasma membrane proteins, such as receptors and ion pumps [98], and/or impairment of mitochondrial function. Mitochondria play a well-recognized role in oxidative stress and apoptosis; in this regard, a key factor in Aβ neurotoxicity could be the opening of permeability transition pores upon Ca^2+^ entry in neuronal mitochondria [99,100], followed by release of strong inducers of apoptosis, such as cytochrome c and apoptosis inducing factor. Intracellular ROS elevation following exposure to amyloid aggregates can also be a consequence of Ca^2+^ entry into cells following non-specific membrane permeabilization. Increased levels of intracellular Ca^2+^ stimulate the oxidative metabolism, providing the ATP needed to support the increased activity of membrane ion pumps involved in clearing the excess Ca^2+^[101]. A similar chain of events could be present in aged tissues, where cells are more susceptible to oxidative stress and their energy load is reduced.

Such a scenario can explain the ROS-Ca^2+^-mitochondrial damage-apoptosis relation described in cells exposed to toxic amyloids [78,98–100]. Indeed, many studies support a close involvement of Ca^2+^ deregulation in AD, PD and prion diseases (reviewed in [101]). As pointed out above, increased intracellular free Ca^2+^ levels can follow any alteration of membrane permeability, possibly resulting from non-specific amyloid pores or other structural modifications provided by cell interaction with aggregates or their misfolded monomers. Membrane lipid peroxidation with production of reactive alkenals and chemical modification of membrane ion pumps can also contribute to the increase of Ca^2+^ levels in aggregate-exposed cells [102].

Similar perturbations of ROS and free Ca^2+^ levels are also found in cells exposed to early aggregates of proteins unrelated to disease; even in this case, cell death by apoptosis or, less frequently, by necrosis is the final outcome [67,103,104]. The biochemical features involved in the different vulnerability of varying cell types exposed to the same toxic pre-fibrillar aggregates have also been studied. Significant correlations between cell resistance, total antioxidant capacity and Ca^2+^-ATPase activity were shown in the investigated cell types [105], further underscoring the importance of early modifications of free Ca^2+^ and redox status as triggers of the chain of events culminating with cell death. Finally, more recent data indicate that the cell membrane biophysical features (rigidity/flexibility, polarity, lateral pressure, *etc.*) resulting from specific lipid composition are very important to determine the extent of cytotoxicity of a given type of aggregate [106].

As outlined before, in most cases, amyloid aggregation implies a first step of aggregate interaction with the plasma membrane. Such an interaction may involve generically the bilayer or, in alternative, proteins, present in specific membrane areas. In the past, several cell surface proteins have been considered as possible candidate receptors for Aβ aggregates, the most investigated in this sense. These receptors could be specific for the shared cross-beta fold rather than for any peculiar structural feature of the Aβ peptides, although, in some cases, they could also be monomer-specific, as in the Aβ-APP, Aβ-TNFR1 or Aβ-PrP interactions proposed to be at the origin of Aβ cytotoxicity [107–109]. Since 1996, the receptor for advanced glycation end products (RAGE) has been proposed as a major candidate as amyloid receptor [110–112]. RAGE transports circulating amyloid-β toxins across the blood-brain barrier (BBB) into the brain, and such an interaction leads to oxidative stress, inflammatory responses and reduced cerebral blood flow (reviewed in [113]). By competing for ligand binding with cell-surface RAGE, its plasma soluble form, sRAGE, might trap circulating ligands preventing their interaction with cell surface receptors. Accordingly, regulating RAGE activity at the BBB and/or increasing plasma sRAGE are considered promising therapeutic targets to prevent vascular damage and neurodegeneration [113,114].

Several other cell surface proteins, including voltage-gated [115] or ligand-gated calcium channels, such as the glutamate NMDA and AMPA receptors [116–118], have also been considered as possible receptors or specific interaction sites for amyloids. In addition, tissue-type plasminogen activator (tPA) has been proposed as a multiligand specific for the cross-β structure [119]. Finally, increasing evidence suggests that additional neuronal binding sites for amyloids, including anionic lipid clusters, such as those provided by the presence of GM1 and other gangliosides in lipid rafts, could be present in the plasma membrane [120]. Such an idea is supported by the finding that any rise of the content of negatively charged lipids results in increased channel formation by amyloids in synthetic lipid bilayers [121]. Overall, the specific effects mediated by any preferential oligomer interaction with membrane proteins could represent a specific therapeutic target in amyloid diseases.

That cell viability is impaired similarly by toxic aggregates grown from peptides and proteins associated or not associated with amyloid diseases supports the idea that, at least in most cases, a common mechanism of cytotoxicity is shared among early aggregates of structurally different peptides and proteins; accordingly, the latter are thought to share common structural features in their pre-fibrillar organization [122]. Although further studies on a wider range of peptides and proteins are needed to explore the generality of these observations, it can be proposed that the cytotoxicity of these types of aggregates is generic [20,123], arising from the “misfolded” nature of the aggregated species and their precursors with exposure of regions (e.g., hydrophobic residues and the polypeptide main-chain), normally buried into the compactly folded native state of the protein. Many of these regions are likely to be aggregation-prone (or “sticky”) and to interact easily with membranes and other cellular components [20,104], thus triggering the complex cascade of biochemical events eventually leading to cell death. Recent data on the variable toxicity of different types of oligomers of the same peptide/protein grown at different conditions, and, hence, with different biophysical features (stability, compactness, hydrophobic exposure), further underscore that oligomer cytotoxicity is strictly dependent on the aggregation conditions and the cell membrane features [106,123].

## 4. Amyloid Aggregation Inhibitors: An Overview

As pointed out above, current research of molecules suitable to counteract amyloid growth and toxicity has shifted from hindering growth and deposition of mature fibrils to avoiding protein misfolding, with the appearance of the most toxic amyloid assemblies arising at the onset of aggregation. The search for treatments able to reinforce the cell defense mechanisms against amyloid cytotoxicity (e.g., the ubiquitin-proteasome and the autophagy-lysosome pathways) is also actively pursued. Compounds able to counteract amyloid aggregation and aggregate toxicity have been particularly investigated for their specific ability (i) to stabilize the amyloidogenic peptides/proteins; (ii) to interfere with the aggregation path preventing the appearance of toxic oligomers; (iii) to inhibit fibril growth and deposition; (iv) to disassemble preformed fibrils; and (v) to inhibit amyloid-membrane interaction. Though the mechanism of action of most potential inhibitors is often still unclear and their effects can vary depending on the conditions in which they are assayed (*i.e.*, target amyloidogenic protein, protein/inhibitor molar ratio, pH, temperature, buffer, interfaces, and so on), efforts have been made to organize the existing knowledge. Accordingly, a classification of amyloid inhibitors into three main categories has been proposed: Class I molecules, inhibiting amyloid oligomerization, but not fibrillization; Class II molecules, inhibiting both oligomerization and fibrillization; and Class III molecules, inhibiting fibrillization, but not oligomerization [55]. This classification does not take into account the mechanism of inhibition; instead, it simply refers to the final outcome of the process. This means that, as we will see in the next sections, different inhibitors can be inserted in the same class though their molecular mechanism of action is substantially different. A large number of natural or synthetic molecules have been investigated for any of these effects, including peptide analogues or variants, peptoids, beta-breaker and cyclic peptides and other peptidic compounds [124–129], antibiotics [130–134], non-steroidal anti-inflammatory compounds [135], antibodies [122,136], many organic substances [137–143] and dyes [144–146], cyclodextrins [147] nanoparticles [148,149], nanomicelles and surfactants [150,151], small molecule colloidal aggregates [152], organofluorine compounds [153], natural phenolic substances (see later) and many others.

Stabilization of peptides and proteins precursors of aggregate growth can be a strategy exploitable at least in some cases. An example is stabilization of transthyretin (TTR) tetramer disfavoring its dissociation in unstable dimers/monomers that can easily undergo aggregation. In this sense, substances that mimic the stabilizing effect of the natural ligand, T4, have been investigated [154–156], and some of them (doxycycline, diflunisal, mds84, tafamidis) have entered clinical trial [157,158]. Another strategy was aimed at finding substances able to disrupt amyloid deposits, as it is the case of several tetracyclines, such as doxycycline [159]; even in this case, clinical trials are underway [160]. Tetracyclines and some analogues may also be useful to inhibit aggregate formation [132,138]. Antibodies may be effective against aggregate nucleation, can stabilize the highly unstable and toxic oligomers, hinder their growth into higher order polymers and mature fibrils and favor aggregate clearance [122,161–163]. These effects are at the basis of the proposed immunotherapy of AD and, possibly, of other diseases [164]. Other inhibitors of amyloid aggregation may act by providing an unfavorable surface in a two-phase system. The latter include nanoparticles with surface chemical properties favoring strong absorption of the peptide/protein [148,149]; colloidal micelles and surfactants may also adsorb strongly monomeric peptides/proteins, avoiding their reciprocal interaction into aggregation nuclei in a way that resembles that displayed by molecular chaperones [150,151]. Curiously, many fibrillization inhibitors recall molecules known to form colloidal particles formed by promiscuous chemical aggregates 50–600 nm in size [165]. Once formed, they physically sequester proteins and inhibit enzymes nonspecifically [165,166]. Like many inhibitors of amyloid polymerization, these colloidal inhibitors are typically highly conjugated, hydrophobic and dye-like, as is the case of Congo red [165], some flavonoids [167] and other organic molecules [168]. The problem with most of the substances listed above is that they cannot be proposed for therapeutic use, and their anti-aggregation effects are merely of scientific interest, providing information to better explain the molecular mechanism of protein/peptide aggregation and to search molecular scaffolds with enhanced anti-amyloid properties.

Molecular mechanisms similar to those listed above also apply for natural phenolic and polyphenolic substances that have been shown to stabilize native states [169,170] or to remodel and inactivate toxic amyloid oligomers by several mechanisms [171–173] that may differ depending on whether they are glycated or not [174]. Natural phenolic substances display at least an advantage over most of the molecules mentioned above: they are of natural origin, are present in many foods and are usually assumed by humans with the diet in variable amounts and more or less continuous way. Such an aspect is of particular interest and may contribute to explain the anti-aging effects of many polyphenols (reviewed in [175]), as well as the results of many epidemiological studies showing a significantly reduced incidence of age-related diseases, particularly neurodegenerative conditions, such as AD and PD, in population with specific dietary regimens, including the so-called Mediterranean diet (MD), where some of these substances are present at relatively high concentrations [176–178].

The following section will revise the most investigated natural phenols and the information gathered in recent years on their effect against amyloid aggregation and their possible use as nutraceuticals or in therapy.

## 5. The Anti-Amyloid Properties of Natural Phenols and Polyphenols

### 5.1. Curcumin

**Figure 1:**
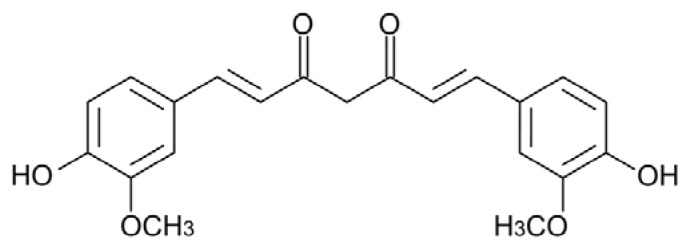
Curcumin structure.

Curcumin (Cur) ((1*E*,6*E*)-1,7-bis(4-hydroxy-3-methoxyphenyl)-1,6-heptadiene-3,5-dione), a diphenol (Figure 1) is the major component of turmeric, a spice derived from the rhizome of *Curcuma longa*.

Turmeric has a long history of use in traditional medicines in China and India [179]; where it is also used as a curry spice in foods. Interestingly, people in India, in spite of a shorter lifetime expectation, present a lower incidence of AD with respect to the US (0.7% *vs.* 3.1% in patients between 70 and 79 year old; [180]), and curry consumption in old age has been associated to better cognitive functions [181].

The extended and conjugated symmetric structure of Cur resembles that of Congo Red, a dye that interferes with the aggregation process [144], binds amyloid aggregates and stains amyloid deposits *in vivo* [182]. After the first evidence of a possible efficacy of Cur against AD coming from a study carried out with the AD transgenic mouse model Tg2576 [183], its power as an inhibitor of amyloid aggregation has been actively investigated *in vitro*. The use of the classical Thioflavin-T (ThT) binding assay to follow the kinetics of amyloid aggregation in the presence of Cur is questionable, though frequently used, due to the superimposition of Cur and ThT fluorescence emission spectra [182] and to the ability of Cur to compete with ThT for fibril binding [184]. Anyway, the efficacy of Cur both as an inhibitor of Aβ amyloid aggregation and as a fibril destabilizer was soon confirmed by transmission electron microscopy (TEM) analysis and dot blot assay of toxic oligomers by using the A11 antibody [182,185]. Subsequently, Cur efficacy as a general inhibitor of amyloid aggregation was tested on several proteins associated with amyloid diseases, unrevealing both common and specific features. In fact, according to the classification proposed by Necula *et al.* [55], Cur works in some cases as a Class I inhibitor (inhibiting amyloid oligomerization, but not fibrillization), in some others as a Class II inhibitor (inhibiting both oligomerization and fibrillization) and, in the case of TTR mutants, as a Class III inhibitor (inhibiting fibrillization, but not oligomerization). In this case, recent work has shown that Cur binds to wt and mutant TTR, increasing its conformational stability both *in vitro* and *ex vivo*, and interferes with the aggregation cascade, redirecting it toward the formation of non-toxic off-pathway intermediates (see below) [186,187].

Cur activity towards Aβ42, hen egg-white lysozyme (HEWL) and human insulin amyloidogenic polypeptide (hIAPP) belongs to the first category of inhibitors: Cur inhibits Aβ42 peptide oligomerization, promoting the deposition of fibrils *in vitro* and *in vivo* [188,189], and counteracts HEWL aggregation, affecting also preformed fibrils, determining, in both cases, the formation of short, sheared fibrillar species [190]. While the fibrillar aggregates that are formed by Aβ and HEWL in the presence of Cur are devoid of cytotoxicity [182,191], this seems not to be the case for hIAPP: circular dichroism (CD) and nuclear magnetic resonance (NMR) analyses reveal that Cur inhibits the structural transition of hIAPP to the α-helical intermediate and slows down the aggregation kinetics, but it neither inhibits the deposition of short fibrils nor disaggregates β-sheet assemblies of hIAPP [192] and, while partially relieving exogenous hIAPP toxicity to INS cells, fails to protect against cytotoxicity in either INS cells overexpressing hIAPP or hIAPP transgenic rat islets [193].

Cur seems to behave like a Class II inhibitor towards α-syn and PrP aggregation. In fact, it increases the solubility of α-syn monomers preventing protein oligomerization dose-dependently and reducing also the amount of high molecular weight aggregates once added to preformed fibrils [194]. In the case of PrP, Cur binds to its acidic α-helical intermediate, to β-sheet oligomers and fibrils (but not to native PrP), as suggested by the increase of the intrinsic fluorescence of Cur in the presence of PrP and by the induced CD spectrum of Cur, reducing the extent of PrP oligomerization and fibrillation [195].

Cur interacts with TTR by competing with T4 for its binding site and stabilizes its tetrameric native state, thus inhibiting fibril formation by TTR aggregation-prone mutants, disaggregating preformed fibrils and determining the formation of small off-pathway non-toxic oligomers, as shown by TEM, dot blot, dynamic light scattering (DLS) analysis and by caspase-3 activity determination in exposed cultured cells [186]. In addition, dietary Cur administration to the hTTR mice model expressing mutant V30M TTR results in potent competition with T4 for TTR binding and in a significant inhibition of tetramer dissociation into non-native monomeric intermediates, decreasing TTR deposition in tissues and reducing Fas-death receptor, ER-resident chaperone BiP and 3-nitrotyrosine levels in the vicinity of the deposits [169]. These properties depict an effect of Cur as a TTR stabilizer, which recalls that of a number of drugs presently under clinical trial (see above).

The observation that the Cur anti-aggregation power results in different outcomes suggests both different molecular mechanisms and common features of inhibition. Evidence suggests that Cur generally binds the target protein/peptide, as shown by the increase of its intrinsic fluorescence in the presence of amyloidogenic proteins [195,196], and by its ability to stain amyloid deposits *in vivo* [182,194], similarly to ThT and Congo Red, even though the fate of the complex varies, depending, in particular, on the initial state of the protein. Cur seems to inhibit both oligomerization and fibrillization when it interacts with the protein monomer in its native form or at the very beginning of its structural transition. In the case of PrP, Cur fluorescence assays reveal that it binds, with a stoichiometry close to 1:1, to the monomeric intermediate, which displays a predominantly α-helical structure and molten globule features, thus preventing its conversion to the amyloidogenic β-sheet rich form. Moreover, the analysis of the CD spectrum of Cur in the presence of preformed PrP oligomers and fibrils suggests that one Cur molecule binds to a PrP dimer (the amyloid building block), possibly intercalating between neighboring PrP subunits, thus preventing fibril growth [195].

In the case of α-syn, the determination by the Trp-Cys contact quenching method of the rate of intramolecular diffusion of the α-syn monomer in the presence or in the absence of Cur provided interesting clues on the mechanism of inhibition of fibril growth [197]. Intramolecular diffusion is the random motion of one part of the protein relative to another. When it is fast, compared to the rate of bimolecular association, aggregation is unlikely, because exposed hydrophobic groups quickly reconfigure, whereas when it is slow, aggregation is favored. Cur binds strongly to the α-syn monomer, with a dissociation constant of 10^−5^ M, and disrupts the long-range interactions inside the peptide chain, increasing the reconfiguration rate of the molecule, thus completely inhibiting both oligomerization and fibrillization.

For what Cur interaction with the Aβ peptide is concerned, its effect on the stability of the Aβ dimer was examined by means of all-atom explicit solvent simulations [198], revealing that Cur acts as an efficient β-sheet breaker, while maintaining the contacts between monomers. This can be the reason why it inhibits the structural transition, leading to the formation of toxic oligomers, but does not avoid protein aggregation. The π-stacking between Cur and Aβ aromatic rings was maintained throughout the whole simulation, suggesting it is an important component of the interaction and a key player of Cur inhibitory activity. The frequent π–π stacking interactions between Cur aromatic rings and the aromatic side chains of His, Tyr and Phe, though transient, would contribute indirectly to a reduction of the β-sheet content in the Aβ dimer. Another computational study suggested that, in Cur, the enolic center and the two phenolic acidic groups separated by a substantially hydrophobic bridge would provide to the molecule both hydrophobic and hydrophilic features; of these, the former could facilitate Cur penetration into the BBB, and the latter could favor subsequent binding to Aβ oligomers [199]. These ideas on the importance of the Cur structural features have been validated by solid state NMR experiments, showing that in Cur, the methoxy and hydroxyl groups adjacent to the aromatic carbons are involved in the Cur-Aβ interaction [200]. These data were confirmed by a study carried out with several Cur synthetic analogs, further suggesting that the carbon spacer between the two aryl rings must contain an enone group to ensure the anti-amyloidogenic activity [201]. The integration of these results with those obtained by Reinke *et al.* [202] by using another library of Cur analogs and the Aβ42 peptide led to hypothesize that a fairly rigid unsaturated, 8–16 Ǻ-long, carbon chain provides the best linker to accommodate the aromatic rings in two distinct (yet unidentified) binding sites on the target peptide.

In spite of its reduced bioavailability (3.6 mmol/kg body weight are required to obtain detectable tissue levels in rats [203]), Cur has been reported to cross the BBB and to label amyloid plaques in AD model mice with various outcomes: in some cases, it reduces plaque burden; in some others, coherently with *in vitro* studies, it reduces A11-positive oligomers, while unaffecting plaque deposition (for a review see [204]). Up until now, clinical trials carried out on humans affected by AD or by mild cognitive impairment have not provided results corroborating Cur efficacy, but several studies are still ongoing (for a review, see [205]).

### 5.2. Epigallocatechin-Gallate

**Figure 2:**
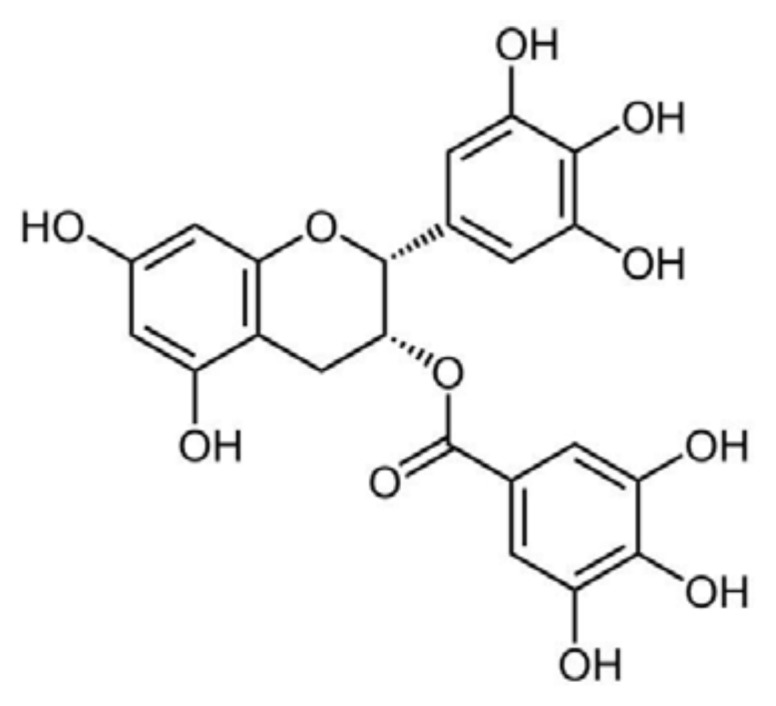
EGCG structure.

(−)-Epigallocatechin-3-gallate (EGCG) (Figure 2) is a member of a family of flavan-3-ols and the most abundant polyphenol in green tea extracts.

Initially, EGCG protection against Aβ toxicity to cultured hippocampal neurons was attributed essentially to its antioxidant activity [206]. Later on, EGCG was shown to reduce both Aβ production in cultured cells and Aβ-amyloid plaque deposition in transgenic mice expressing the “Swedish” APP mutant, by promoting the non-amyloidogenic α-secretase proteolytic pathway [207]. A study aimed at investigating the association between green tea consumption and cognitive function in elderly Japanese subjects showed that a higher consumption of this beverage was associated with a lower prevalence of cognitive impairment in humans [208]. A contribution of the EGCG iron chelating property, resulting in modulation of APP translation via an IRE (IRE type II) present in the 5′-UTR region of APP, was also considered of importance [209]. At the same time, EGCG was shown to interfere directly with amyloid fibril formation from several peptides and proteins and to remodel preformed fibrils, thus generating non-toxic species. The investigated peptides/proteins include Aβ, α-syn, mutant htt, TTR, hIAPP, the amyloidogenic peptide PAP_248–286_ from prostatic acidic phosphatase (that aggregates into fibrils known as semen-derived enhancer of virus infection–SEVI), HEWL, k-casein and calcitonin [210–218].

The study of the molecular features of EGCG interaction with amyloidogenic peptides/proteins disclosed two different modalities that also corresponded to different outcomes in terms of inhibitory activity and final products: (i) EGCG binds to the unfolded or misfolded peptide/protein with non-covalent random interactions involving the peptide backbone and redirects aggregation towards the formation of small off-pathway oligomers or amorphous material; and (ii) EGCG binds to the native form of the peptide/protein through interactions with the side-chains of specific residues that maintain peptide/protein native configuration preventing its aggregation. The first pattern of inhibitory activity is displayed by EGCG towards α-syn, Aβ, htt, hIAPP and HEWL [212,219–221]. In the case of α-syn and Aβ, EGCG stimulates the assembly of amorphous material and of spherical SDS-resistant oligomers that are not recognized by the A11 antibody and have no seeding properties; for these reasons, these assemblies are considered off-pathway with respect to the fibrillization process [219]. EGCG also remodels Aβ and α-syn mature fibrils and toxic oligomers into non-toxic small protein aggregates, without fibril depolymerization, determining a loss of β-sheet content [222]. This implies that EGCG binds to unfolded monomers, oligomers and fibrils, as confirmed by the nitro blue tetrazolium (NBT) staining assay. It was initially suggested that EGCG inhibits the conformational transition of Aβ from a random coil to the amyloidogenic β-sheet structure, thereby favoring the deposition of unstructured aggregates [219]; however, recent solid-state NMR analysis indicates that the small oligomers formed in the presence of EGCG show defined correlation signals, suggesting that they are not amorphous, but significantly structured, though unable to seed further fibril growth and devoid of toxicity [223]. A very recent study conducted on Aβ40, hIAPP and the Sup35NM_Ac7–16_ Y→F mutant showed that hydrophobic interaction of the oxidized form of the polyphenol with the peptide is mainly responsible for EGCG remodeling activity [224]; when free amines or thiols are proximal to the EGCG hydrophobic binding sites, the EGCG-based quinones are then capable of covalently modifying the amyloidogenic proteins through Schiff base formation, but this is not a pre-requisite for amyloid remodeling.

The thermodynamics of the EGCG-Aβ interaction were studied by isothermal titration calorimetry [225]; in the 25–45 °C range and at EGCG:Aβ ratios <16, such an interaction is enthalpy-driven and, hence, mainly dependent on hydrogen bonding (hydrophobic interactions are entropy-driven, but entropy decreases during the process, so they do not seem to be involved). EGCG-Aβ interaction is favored by pH values either lower or higher than the Aβ pI (pH 5.0): at both conditions the formation of hydrogen bonds between EGCG and Aβ is favored. Thermodynamic analysis further reveals that when the EGCG:Aβ ratio is in the 16–46 range; the interaction is favored by both a decrease of enthalpy and an increase of entropy, indicating important roles of both hydrogen bonding and hydrophobic interactions. When the EGCG:Aβ ratio rises over 46, entropy becomes the only driving force, suggesting a predominant contribution of the hydrophobic interactions. The ΔG values at different solution conditions are essentially invariant, since the hydrogen bonding (mainly involving the Aβ1–16 region) and the hydrophobic interaction (mainly involving the Aβ17–42 region) change in opposite ways, compensating for each other and ensuring EGCG binding to Aβ over a broad range of solution conditions [226].

The predominance of the interaction of EGCG with the peptide backbone characterizes its action on hIAPP. EGCG was found to be still effective in inhibiting the aggregation of hIAPP mutants lacking the best candidate sites for interaction with a polyphenolic ligand, namely the three conserved aromatic residues, the disulfide group and the free amino group. By a process of elimination, the authors concluded that EGCG interacts with hIAPP by hydrogen bonding to the peptide backbone and by relatively non-specific (presumably hydrophobic) interactions of the gallate ester with side chains [220]. The fundamental role of the gallate ester in the anti-aggregation power of EGCG was further confirmed by showing that (−)-gallocatechin, (−)-epigallocatechin and epicatechin display reduced efficacy (or total inefficacy) against htt, Aβ, α-syn, HEWL and hIAPP aggregation [216,222,227].

The importance of hydrophobic interactions in the inhibitory activity of EGCG towards hIAPP aggregation was confirmed by both Palhano *et al.* [224] and Engel *et al.* [228], but while the former showed that the polyphenol was still able to disaggregate hIAPP fibrils in the presence of detergent micelles, the latter demonstrated that in the presence of a DPPG monolayer EGCG anti-aggregating activity towards hIAPP was much reduced and its ability to disaggregate preformed fibrils completely suppressed, due to the polyphenol inability to access the binding sites on the peptide and, possibly, to its sequestration by the lipid interface [228]. In light of the importance of membrane damage in the context of amyloid toxicity and considering the general ability of polyphenols to interact with membranes, thus changing their physical properties [229,230], this topic deserves further investigation, using standardized experimental conditions.

EGCG inhibits TTR, SEVI and k-casein aggregation by stabilizing their native conformations [217,222,231]. This was confirmed for TTR by treating with EGCG a familial amyloidotic polyneuropathy (FAP) mouse model expressing human V30M TTR. In this model, an increase of serum TTR tetramer stability was observed, accompanied by a reduction of TTR congophilic deposits [168,186]. *In vitro* studies showed that EGCG binds to both wt and mutant TTR, as it is evident by NBT staining, forming an SDS-stable adduct with a 1:1 molar ratio, as confirmed by MS analysis. Such interaction does not involve the T4 binding site and stabilizes the native tetramer, thus inhibiting aggregation. EGCG is also able to destabilize preformed TTR fibrils, generating small polydispersed amorphous aggregates [186,222]. The crystal structure of the EGCG-V30M TTR complex has shown that EGCG binds specific residues at the interface between two TTR monomers, thus stabilizing the tetramer. EGCG can also induce the association of two TTR tetramers (both wt and mutant) into small oligomers that are non-toxic to IMR-32 neuroblastoma cells [232]. Even in this case, similarly to Cur, EGCG appears to behave in a way that recalls that of drugs currently under clinical trials as TTR stabilizers (see above).

The association between EGCG and PAP_248–286_ (the prostatic acid phosphatase fragment that aggregates into SEVI fibrils [231]) is also characterized by the interaction with specific amino acid residues; the interaction inhibits fibril formation and leads to fibril depolymerization both at neutral and mildly acidic pH (typical of vaginal fluid). NMR and electrospray ionization-mass spectrometry (ESI-MS) analysis together with the NBT assay suggested that EGCG binds monomeric PAP_248–286_ through a multistep process: initially, a weakly bound noncovalent complex is formed, where most likely hydrogen bonding and hydrophobic interactions predominate; subsequently, after the generation of a reactive quinone complex by EGCG auto-oxidation, a covalent complex arises through Schiff base addition involving lysine side chains [231].

RCMκ-CN (reduced and carboxymethylated κ-casein), a non-disease-associated natively-disordered amyloidogenic protein, was used as a peptide model to investigate the EGCG anti-aggregation activity. Although predominantly unfolded, RCMκ-CN contains a large antiparallel β-sheet region that requires little conformational change to aggregate, forming the cross-β-sheet core of mature RCMκ-CN amyloid fibrils. Furthermore, in this case, EGCG (in a 1:1 molar ratio with RCMκ-CN) was shown to constrain the protein in its micellar native-like state, rather than redirecting its aggregation path towards the formation of amorphous deposits [217]. NMR experiments demonstrated that EGCG suppresses RCMκ-CN fibril formation by interacting with its relatively rigid amyloidogenic β-sheet region through strong SDS-stable hydrophobic associations and, possibly, π–π stacking interactions with aromatic residues.

For what the possible pharmacological use of EGCG is concerned, most of the ingested EGCG does not get into the blood: absorption takes place in the small intestine, but substantial quantities pass to the large intestine to be eliminated [233]. The conditions that seem to increase EGCG plasma levels are administration after an overnight fasting period together with 200 mg ascorbic acid and 1,000 mg salmon omega-3 fatty acids, without caffeine [234]. Omega-3 polyunsaturated fatty acids seems to enhance oral bioavailability of EGCG and also to improve its efficacy [235].

### 5.3. Resveratrol

**Figure 3:**
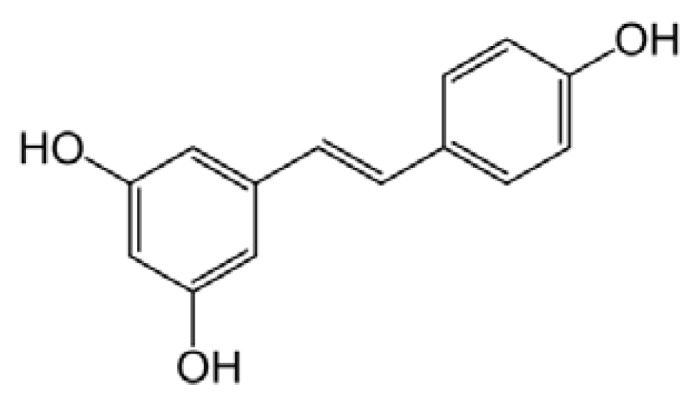
Resveratrol structure.

Resveratrol (3,5,4′-trihydroxystilbene) is a non-flavonoid polyphenol that occurs in several foods and is particularly abundant in the skin and seeds of the grape’s fruit (*Vitis vinifera*) and in red wine (Figure 3).

Resveratrol is a member of the stilbene family, a group of compounds that consist of two aromatic rings joined by a methylene bridge. It has attracted great attention, since it was considered responsible for the cardioprotective effects of red wine, resulting in a low incidence of cardiovascular diseases in the French population despite a diet rich in saturated fats (the so-called “French Paradox”). Such protection comes from the ability of red wine and grape extract to reduce platelet aggregation, uphold vasorelaxation, prevent atherosclerosis, decrease lipid peroxidation and ameliorate serum cholesterol and triglyceride concentrations [236].

Since the first study, published in 1997, reporting that moderate-to-mild wine consumption was associated with a low risk of AD [237], both epidemiologic studies and experimental treatment of animal models have shown that resveratrol may be a powerful agent preventing β-amyloid deposition-associated neurodegeneration [238–240]. Accordingly, several attempts are being made to design resveratrol derivatives with enhanced protective properties [241] and to improve its bioavailability, for example, by using lipid-core nanocapsules [242,243]. The biochemical mechanism of resveratrol protection is multifaceted; besides its antioxidant activity [244], it involves modulatory effects on several pathways that were investigated particularly in the context of Aβ toxicity. It was shown to reduce both intracellular and secreted Aβ in cell culture by promoting its proteasome-dependent degradation [245]; to reduce Aβ-stimulated death signals, such as NF-κB signaling and p53 activity, by activating SIRT-1 and mimicking the effect of caloric restriction [246,247]; and to increase AMPK activity, thus triggering the autophagic degradation of Aβ aggregates [248]. These (and other) pieces of evidence support the hypothesis that resveratrol acts upstream of one (or more) signaling cascades, possibly triggered upon binding to a membrane receptor. Actually, potential binding sites widely distributed in rat brain were detected in the plasma membrane and, to a lesser extent, in nuclear and cellular fractions of rat brain homogenates [249].

Most notably, resveratrol is also able to directly interfere with the amyloid aggregation of different peptides, reducing their toxicity, as is the case for hIAPP [250]. By X-ray reflectivity measurements, it was also shown that resveratrol inhibits hIAPP interaction with a lipid layer interacting preferentially with peptide monomers [251], a particularly valuable effect, considering that hIAPP fibrillization at the cell membrane and subsequent membrane destabilization are important determinants of hIAPP toxicity [252]. Fluorescence microscopy analysis further suggested that resveratrol redirects hIAPP aggregation towards the formation of amorphous non-toxic aggregates that do not interact with INS-1E cells [253], and following NMR analysis, it was proposed that resveratrol binds to hIAPP His-18, thereby inhibiting early formation of oligomeric intermediates [254].

Resveratrol was shown to dose-dependently inhibit the Aβ peptide fibrillization and to depolymerize its preformed fibrils, in both cases leading to the formation of non-toxic oligomers [255]. Actually, resveratrol seems to remodel a subset of Aβ conformers that possess random coil (A11-positive soluble oligomers, but not non-toxic oligomers) or β-sheet (OC-positive fibrillar intermediates and fibrils) structures into SDS-resistant, non-toxic, unstructured conformers [172]. The direct interaction of resveratrol with Aβ monomers and fibrils was confirmed by surface plasmon resonance (SPR) and NMR analysis [256].

X-ray structure analysis showed that resveratrol forms a complex with TTR, where it fits the T4 binding site by non-polar contacts mediated by the stilbene moiety, reinforced by hydrogen bonds involving its hydroxyl groups, thus preventing protein aggregation [257].

Resveratrol can also inhibit lysozyme aggregation and depolymerize pre-formed fibrils [258]. An interesting study carried out on this polyphenol (and on a panel of different phenolic substances) showed that its glycosidic and aglycone forms remodel A11-positive Aβ oligomers and OC-positive fibrils through different pathways: while the aglycone remodels the oligomers into large aggregates, the glycoside (named piceid) disaggregates them, releasing the soluble peptide. From these results, it was hypothesized that the aglycone moiety of polyphenols disrupts those intermolecular contacts that involve aromatic groups, leading the newly exposed aromatic residues to associate with the sugar moiety (when present); accordingly, other intra- and inter-molecular associations are prevented, and the formation of amorphous aggregates is avoided. Structure comparison analysis suggest that, in polyphenols, the number of phenolic rings is the primary determinant of the remodeling efficacy and that the minimal structural requirement for phenolic aglycones to remodel Aβ oligomers consists in two aromatic rings, of which at least one must possess an hydroxyl moiety [174].

### 5.4. Quercetin and Myricetin

**Figure 4:**
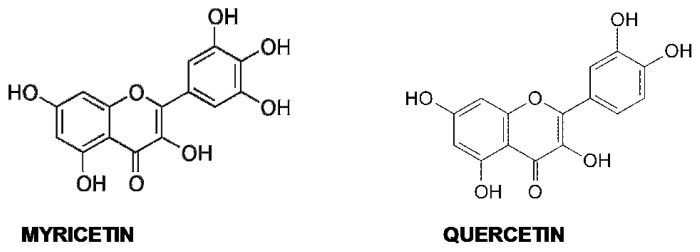
Myricetin and quercetin structure.

Quercetin (3,30,40,5,7-penta-hydroxyflavone) and myricetin (3,3′,4,5,5′,7-hexahydroxyflavone) (Figure 4) are flavonols found in many foods of vegetal origin, including tea, onions, cocoa and red wine, and in *Ginkgo biloba* EGb 761 extract.

Unlike the phenols described in the previous paragraphs, these substances were initially investigated for their anti-amyloidogenic activity *in vitro* and only subsequently tested for their efficacy *in vivo*. By ThT binding and TEM analysis, they were shown to prevent the growth of Aβ amyloid aggregates *in vitro* and to destabilize preformed fibrils [185]. In a study carried out on 39 flavonoids, the strong inhibitory effects against Aβ fibrillation of the flavones were outlined, and quercetin was shown to be one of the most potent representatives of this category (IC_50_ = 2.4 μg/mL), providing also cell protection against Aβ cytotoxicity [259]. It was suggested that the 3-hydroxy, 4-keto groups of flavonols are essential for inhibition of Aβ fibril growth, but a subsequent study reached different conclusions, showing that the 3-hydroxyl group is not necessary, while the 3′,4′-dihydroxyl group of the B ring is essential [260]. Whatever the case, there is consensus on the assumption that increasing the number of hydroxyl groups on the B ring improves the anti-aggregation effect of flavonoids; in fact, myricetin, which contains an additional hydroxyl group, is more effective than quercetin against Aβ aggregation [261]. Quercetin (like other flavonols) was also shown to heavily quench ThT fluorescence emission [184], so all the conclusions concerning the *in vitro* anti-amyloidogenic activity of flavonols must be supported by further experimental pieces of evidence.

Quercetin and myricetin efficacy as amyloid aggregation inhibitors and preformed fibril destabilizers was confirmed with insulin and α-syn, respectively [262,263]; yet, other compelling results were also reported: Jagota *et al.* [264] suggested that quercetin inhibits Aβ fibrillization, but not its toxic oligomerization, hence increasing Aβ toxicity in a *C. elegans* model of Aβ deposition; Noor *et al.* [265] reported that quercetin and myricetin were not able to prevent hIAPP fibrillation, at variance with morin (which differs only for the position and/or the number of the hydroxyl groups on the B ring). However, they did not investigate the possible effects of the two flavonols either on the most harmful oligomeric species or on aggregate toxicity. Actually, myricetin was later shown to dose-dependently inhibit hIAPP toxic aggregation [266]. The way both flavonols inhibit amyloid aggregation is still a matter of debate, as well, even considering their activity on the same amyloidogenic peptide. In fact, data obtained by SPR analysis and fluorescence spectroscopy of the compounds in their free or bound state led to speculate that they would preferentially bind to Aβ fibrils, not to monomers, particularly to the fibril growing ends, competing with monomers for binding and inhibiting fibril elongation, behaving as Class II inhibitors [267]; the opposite was found, by treating the Tg2576 mice model of AD with myricetin, where a decrease of A11-positive oligomers with no change of plaque burden was observed, suggesting that, rather, it would act as a class I inhibitor. The data agree with an *in vitro* study showing that myricetin could hamper Aβ oligomerization, but not fibrillization [55,188]. Finally, electrospray ionization-ion trap mass spectrometry (ESI-IT MS) analysis of Aβ monomers revealed their prolonged persistence in the presence of myricetin, even at aggregation conditions, indicating that myricetin targets preferentially Aβ monomers and short transient oligomers, thus lowering the aggregation rate and reducing fibrillization, also. These data support the idea that myricetin behaves as a Class III inhibitor [268]. Moreover, other MS analysis suggested that myricetin could exert a pro-oxidant activity on Aβ Met^35^, hindering the amyloidogenic conformation [269]. However, this assumption was not validated by NMR studies, which confirmed preferential binding of myricetin to localized regions of monomeric Aβ, hindering oligomerization and reducing synaptoxicity [270].

Overall, these and other data indicate that, at present, there is not a consensus on the molecular features of the myricetin anti-amyloidogenic power. However, the subject is being actively investigated; computational analysis performed on Aβ GNNQQNY fragment oligomers suggested that myricetin forms H-bonds with the surface of the β-sheet, weakening the interstrand hydrogen bonds and disaggregating the outer layer of the aggregate [271], while CD and deep-ultraviolet resonance Raman spectroscopy supported the importance of the aromatic interactions [272].

Besides a direct inhibitory effect on amyloid aggregation, quercetin and myricetin seem to exert a general protection against amyloid-induced cytotoxicity acting at multiple levels. In fact, they inhibit β-site APP cleaving enzyme-1 (BACE-1) activity [273], scavenge ROS [274–276] and also behave as competitive inhibitors against the oxidative activity of the complex Aβ-Cu^2+^ by interacting with the metal binding site on Aβ via their α-keto enolate group, thus maintaining the integrity of their catechol moiety with a resulting bifunctional inhibitory activity (metal chelation and Aβ interaction) against amyloid aggregation [277,278].

Quercetin also activates AMPK, thus triggering a pleiotropic neuroprotective cascade [279], enhances neurogenesis and synaptogenesis through a CREB phosphorylation-mediated signaling pathway [280] and hinders the interaction of amyloidogenic peptides with membranes and their permeabilization by inserting between the outer part of the hydrophobic core and the external hydrophilic layer of the lipid bilayer [281]. Finally, myricetin relieves glutamate-induced neuronal toxicity by multiple pathways (*N*-methyl-d-aspartate receptor modulation and reduction of intracellular Ca^2+^ overload, inhibition of ROS production, reduction of caspase-3 activation) [282] and inhibits Tau filament formation [283].

### 5.5. Olive Oil Phenols

Olive oil phenols have attracted more and more attention since, together with resveratrol (see above) and other phenols, they are typical components of the so-called MD that, among others, is believed to provide significant protection against mild cognitive impairment and its conversion to AD [176]. Studies in rodents suggest that diet supplementation with extra virgin olive oil (EVOO) improves learning and behavioral deficits associated with aging and disease [284,285]. In addition, several reports, including the “Three city study” [286], support a strict association between many protective effects of the MD and the sustained assumption of EVOO. The phenols most abundant in the EVOO and most investigated for their ability to prevent amyloid aggregation and its toxic effects both *in vitro* and *in vivo* are oleuropein aglycone and oleocanthal.

**Figure 5:**
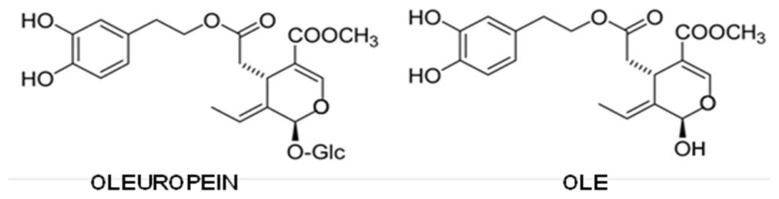
Oleuropein and oleuropein aglycone (OLE) structures.

Oleuropein is a secoiridoid and can be considered the ester between hydroxytyrosol and the glucosidic form of elenolic acid. It is deglycosylated by a β-glucosidase released from olive fruits during crushing, giving rise to oleuropein aglycone (OLE) (Figure 5), which, due to its high hydrophobicity, is retrieved in oil [287].

The content of OLE in olive drupe depends on the cultivar [288,289] and the time of ripening; in addition, the recovery of the deglycosylated oleuropein derivative depends on the way the fruits are processed to obtain EVOO [290]. Finally, the content of OLE in EVOO depends on oil ageing, as the molecule undergoes degradation (mainly oxidation) with time [291].

Oleuropein can associate to the monomeric form of Aβ40 and AβMet^35^(O) with a 1:1–2:1 stoichiometry; such an interaction is non-covalent, but possesses a remarkable binding energy, since the complexes are still observable when an orifice potential of 100 V is applied to the ESI-MS apparatus [292,293]. Through enzymatic cleavage of the Aβ:oleuropein complex prior to ESI-MS analysis, three peptide segments were identified as being implicated in the interaction; of these, the hydrophobic (17–21) one is the best candidate to interact with the non-polar moiety of oleuropein [293] and, hence, also with its aglycone derivative. Interestingly, the Aβ sequence critical for peptide fibrillization overlaps the putative OLE binding region [294–297]. The interaction of oleuropein with Aβ monomers, and the importance of the 17–21 region for it were further confirmed by NMR [297,298]; a possible interaction of the secoiridoid also with Aβ oligomers cannot be excluded by this technique.

Most of the research on oleuropein as an aggregation inhibitor was performed on its aglycone derivative, which was shown to interfere with both hIAPP and Aβ42 aggregation [299,300]. Structural analysis (ThT and Anilinonaphthalene-8-sulfonate binding, CD, Atomic Force Microscopy, TEM) showed that OLE does not suppress the final growth of mature amyloid fibrils; rather, it triggers peptide precipitation into amorphous aggregates devoid of toxicity, avoiding the formation of toxic oligomers. This amorphous precipitate eventually evolves into non-harmful protofibrils, leading to the ranking of OLE as a Class I inhibitor. For what hIAPP aggregation is concerned, the amorphous aggregates that originate during the first phases of incubation in the presence of OLE were shown to be unable to interact with, and to damage, the cell membrane [299]. Aβ fibrils can also be remodeled by this secoiridoid without release of toxic fragments [300].

Finally, recent data indicate that OLE can be protective against amyloid aggregation and aggregate toxicity not only to cultured cells, but also *in vivo*. In fact, its administration to a *C. elegans* strain expressing Aβ42 resulted in significant reduction of plaque deposition and toxic oligomer formation and improvement of worm performance upon reduction of the extent of paralysis and increase of survival [301]. Finally, we recently showed that dietary supplementation of OLE strongly improved the cognitive performance of the TgCRND8 mouse model of AD; mice showed remarkably reduced plaque deposits, microglia migration to the plaques for phagocytosis, a reduction of the astrocyte reaction and an astonishingly intense autophagic reaction. Data obtained with cultured cells confirmed that OLE is able to induce autophagy, possibly by acting on the mTOR pathway [302]. OLE protection did not merely result from its power as an antioxidant, neither in cells nor in worms [299,303], and was more evident when the compound was administered at the beginning of the aggregation process.

OLE was more effective than oleuropein and hydroxytyrosol (that, anyway, were also active) as an inhibitor of Tau fibrillization, with an IC_50_ of 1.4 μM, as ascertained by both DLS and ThS fluorescence assay [304]. While OLE and oleuropein led to a scarce deposition of fibrillar material in the form of short rods, hydroxytyrosol induced the growth of fibrils that were less dense, but similar to those grown in the absence of any inhibitor. Oleuropein was also shown to modify APP processing, increasing the formation of the non-amyloidogenic and neuroprotective sAPPα fragment and to decrease Aβ oligomers in HEK695 cell supernatants by increasing matrix metalloproteinase-9 (MMP-9) secretion [305].

**Figure 6:**
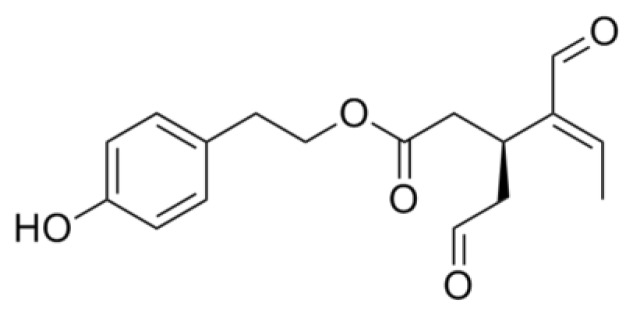
Oleocanthal structure.

Oleocanthal is the dialdehydic form of (−)-deacetoxy-ligstroside aglycone (Figure 6).

It was shown to bind to monomeric or oligomeric Aβ42, inducing the growth of higher molecular weight aggregates that were more immunoreactive and less toxic to hippocampal neurons than their original counterparts; furthermore, maintaining neurons in oleocanthal for even a short period before exposure to toxic oligomers resulted in an effective reduction of their binding to the cell membrane [305]. In the same year, oleocanthal was shown to inhibit Tau fibrillization by ThT, EM, SPR and MALDI-TOF MS [306]. Most likely, such an inhibition involved chemical crosslinking of Tau by reaction of the oleocanthal dialdehydic group with lysine residues, thus arresting the conversion of Tau from random coil to β-sheet and promoting an increase of α-helical structure [307]. As already observed for Aβ, also in this case, oleocanthal determined the conversion of monomers and oligomers to high molecular weight aggregates, suggesting that crosslinking may be the typical mechanism of action of this phenol. Actually, a structure-activity relationship study using several oleocanthal analogues clearly indicated that the two aldehydic groups of oleocanthal are essential to prevent Tau fibrillogenesis. Interestingly, oleocanthal reveals a certain degree of substrate specificity, showing a low reactivity toward free nucleophilic amino acids, such as lysine and arginine, and no binding to several other proteins. Studies with transgenic mice recently revealed that this phenol enhances β-amyloid clearance from the brain by upregulating Aβ transport proteins at the BBB and Aβ degrading enzymes [308].

A comparison of the very similar chemical structures of OLE and oleocanthal reveals that the main difference between them is the presence, in the former, of a methoxycarbonyl group, which dramatically increases the acidity of the hydrogen on the adjacent carbon, leading to intramolecular rearrangements eventually culminating in the conversion to the dihydropyran form, the main isomer of OLE in solution. As a direct consequence, OLE has only one aldehyde function and, so, cannot work as a chemical crosslinker, as is the case for oleocanthal [303]. In rat and humans, orally administered olive oil phenols, including OLE, its glycoside and/or one of its derivatives arising from tissue metabolism are intestinally absorbed, skipping degradation by microorganisms in the colon, cross the blood-brain barrier and are found inside brain parenchyma [309,310].

### 5.6. Other Phenols

**Figure 7:**
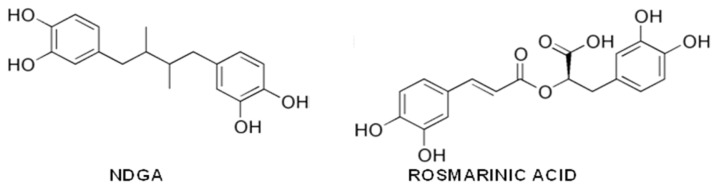
Nordihydroguaiaretic acid (NDGA) and rosmarinic acid structures.

Among the other natural phenols that have attracted the researchers’ attention, the stilbene, nordihydroguaiaretic acid (NDGA), a potent anti-oxidant found in the long-lived creosote bush, and the phenolic acid rosmarinic acid (Figure 7), present in may culinary herbs, such as rosemary, oregano, sage, thyme and peppermint, were the most investigated, but the results concerning their efficacy were not always clear.

For example, NDGA was first shown to depolymerize preformed Aβ fibrils without release of toxic species [311]. This result was subsequently questioned, and it was suggested that, while not disaggregating Aβ fibrils or altering protofibril elongation, NDGA would inhibit protofibril-protofibril association, possibly by binding along their lateral surface, thus reducing cytotoxicity [312]. Later on, a study on the Tg2576 mice model of AD showed that both NDGA and rosmarinic acid reduced Aβ plaque burden, but while the latter inhibited the aggregation pathway from Aβ monomers to A11-positive oligomers and from these to Aβ fibrils, NDGA mainly inhibited the pathway from A11-positive oligomers to Aβ fibrils without interfering with the growth of toxic oligomers [189]. This is the contrary of what was previously demonstrated *in vitro*, where NDGA inhibited Aβ oligomerization, but did not affect its fibrillization [55]. Finally, it was recently shown by PICUP detection of oligomers and NMR analysis that NDGA (similarly to myricetin, ferulic acid and Cur) binds to specific peptide regions of Aβ monomers, while rosmarinic acid interacts only with oligomers, and all of them prevent both oligomerization and fibrillization reducing neuronal toxicity. The authors speculated that the negative results reported for NDGA in the mice model by Hamaguchi *et al.* [188] would derive from a low specificity of the A11 antibody in recognizing toxic oligomers [270]. NDGA was only slightly effective in reducing TTR aggregation [186], so its efficacy is still a matter of debate.

The array of natural phenols being tested as potential inhibitors of amyloid aggregation becomes wider and wider every day and now includes also tannic acid [207,313] and the product of tannin hydrolysis ellagic acid [314]; several flavones, like apigenin [315,316], baicalein [315,317,318], kaempferol [260,261,263,264,271,319], morin [265,320,321], fisetin [259,260,321], rutin (the glycoside of quercetin with the disaccharide rutinose) [322,323] and luteolin [260,324]; rottlerin, already known as a PKC-δ inhibitor [325,326]; the anthocyanidin malvidin, the main responsible for the red color of wine [327]; the stilbenes piceatannol [328] and the resveratrol-derived dimer ɛ-viniferin glucoside [329]; and, finally, ferulic acid (related to trans-cinnamic acid) [189,263,264,330–332].

## 6. Conclusions

The last 15 years have seen a huge rise of the interest in degenerative diseases with protein deposits, of which amyloid diseases are by far the most important. In particular, the increasing rise of aged people in modern countries has stressed what is considered the major risk factor of the sporadic forms of these diseases: aging. Presently, AD, PD and type 2 diabetes pose terrific socio-sanitary problems to an increasing number of families in the US, Europe, Australia and Japan, together with an unsustainable burden to the national health services. The problem is made even worst by the general consensus that the occurrence of these pathologies will further increase in the near future. This is why increasing efforts are being made to elucidate the molecular basis of protein aggregation into amyloid assemblies, both *in vitro* and in tissue, and the effects of these assemblies on living systems, from the simplest ones (cultured cells) to animal models of these pathologies and, eventually, to humans.

In spite of the steps forward to a better understanding of the molecular pathogenesis of amyloid diseases performed in recent years, much must still be learned and a deeper knowledge appears mandatory to develop therapeutic approaches to these diseases, which are presently lacking. However, in parallel with many studies on the molecular basis of protein aggregation and aggregate toxicity, many efforts have been spent, and are presently under way, to test and to explain, when present, the anti-aggregation and cell protective power of a wide panel of natural or synthetic chemicals, some of which are already in use as antibiotics or other drugs. The problem with most of these chemicals is that they are not suitable for long-term administration in possibly high doses; other problems are associated with the way of administration and their possible side-effects, as it was found for immunological therapy. Nevertheless, research is going on, and it is expected that some of these chemicals, particularly the antibodies raised by passive or active immunotherapy, will become effective therapeutic tools without significant side-effects.

The very complex picture of the biological determinants of protein aggregation, the interplay between the structural features underlying the cytotoxicity of specific amyloid assemblies and of cell vulnerability to their toxicity, the specific cell/tissue functionally aberrant responses to the presence of amyloid deposits, the biological tools protecting cells against amyloid toxicity, and many others, make it very difficult to find specific drugs with effective therapeutic potential. These considerations are further stressed by the fact that in most amyloid diseases, the appearance of early symptoms signals a condition where cell/tissue damage is too severe to be reversed. However, an alternative possibility is provided by molecules whose continued administration is able to counteract any of these aberrant situations, so as to reduce the extent of cell/tissue insult or to delay it significantly. It is evident that delaying the appearance of the symptoms of age-associated conditions, such as AD or PD, would greatly reduce the prevalence of the latter in the aged population.

Many natural phenolic substances occur in a vast array of vegetables, herbs and fruits, some of which are important components of the diet of specific populations: this is the case for East Asian spices and herbs enriched in Cur and EGCG or the MD in a number of countries lining the Mediterranean sea, characterized by the use of EVOO and red wine, whose phenolic substances (resveratrol, oleuropein aglycone, oleocanthal) are continuously taken up in variable, yet significant, amounts with the diet. Increasing molecular, biochemical, biological and clinical information support the notion that at least some of these substances possess beneficial properties that are associated not only with a reduction of the aggregation power of specific peptides/proteins associated with amyloid diseases, but also with the ability to stimulate cell defenses against the insult given by these aggregates, thus providing a convincing explanation of the recognized protection of these diets against a number of pathological conditions, including aging and age-associated pathologies, such as PD and dementia. These substances can be seen as nutraceuticals rather than drugs and can be administered in larger amounts than specific drugs; moreover, the possibility to fortify diet components, such as wine and olive oil, with these substances could be a way to take them up for long time periods, replenishing possible deposits in the organism (most of them are hydrophobic molecules that, similarly to lipid vitamins, could be stored in the adipose tissue), possibly providing continuous protection and a way to delay aging, dementia and other neurodegenerative diseases. More information is needed for the possible use of any of these compounds in humans, and convincing clinical trials are still to be carried out. Nevertheless, these substances undoubtedly represent molecules with high potential as effective therapeutic/prophylactic tools to treat amyloid diseases.

These natural compounds can also be regarded as tools to investigate the amyloid aggregation pathway and as molecular scaffolds for the development of more active and biologically available drugs. In this respect, a major problem that emerges from this review is the absence of a rigorously defined methodological and technical framework to which the researchers, willing to investigate this peculiar class of amyloid inhibitors, should adapt. As we have outlined, some widely used assays suffer from an interference of phenolics, which is not always adequately taken into account; on top of this, a clear definition of what is intended for “oligomers”, “pre-fibrillar aggregates” and “fibrils” is still lacking. The need to standardize the *in vitro* and *in vivo* assay conditions is compelling in order to rigorously determine the efficacy, specificity and potency of these promising inhibitors.
